# What does population structure analysis reveal about the *Pterostylis longifolia* complex (Orchidaceae)?

**DOI:** 10.1002/ece3.376

**Published:** 2012-09-21

**Authors:** Jasmine K Janes, Dorothy A Steane, René E Vaillancourt

**Affiliations:** 1 School of Plant Science, University of TasmaniaPrivate Bag 55, Hobart, Tasmania, 7001, Australia; 2 Biological Sciences, University of AlbertaEdmonton, Alberta, T6G 2E9, Canada

**Keywords:** AFLP, conservation, hybridization, refugia, speciation, taxonomy

## Abstract

Morphologically similar groups of species are common and pose significant challenges for taxonomists. Differences in approaches to classifying unique species can result in some species being overlooked, whereas others are wrongly conserved. The genetic diversity and population structure of the *Pterostylis longifolia* complex (Orchidaceae) in Tasmania was investigated to determine if four species, and potential hybrids, could be distinguished through genomic AFLP and chloroplast restriction-fragment-length polymorphism (RFLP) markers. Analysis of molecular variance (AMOVA) results indicated that little genetic variation was present among taxa, whereas PCoA analyses revealed genetic variation at a regional scale irrespective of taxa. Population genetic structure analyses identified three clusters that correspond to regional genetic and single taxon-specific phenotypic variation. The results from this study suggest that “longifolia” species have persisted throughout the last glacial maximum in Tasmania and that the complex may be best treated as a single taxon with several morphotypes. These results could have serious evolutionary and conservation implications as taxonomic changes could result in the instatement of a single, widespread taxon in which rarer morphotypes are not protected.

## Introduction

Speciation is the innovative process leading to the creation of new species. Understanding the general patterns and processes of speciation is essential in explaining the diversity of life (Barraclough and Nee 2001). Taxonomy, the science of classification of species, is a challenging field that relies heavily on specialists that possess exceptionally intimate knowledge of the organisms with which they work (Tautz et al. 2003). Our desire for resolute order stems not only from curiosity, but from a need to have a basic unit that can be applied to measures of evolution, biodiversity, conservation, and taxonomy. However, conflicting theoretical views and methodological approaches (Charles and Godfray 2002) have resulted in taxonomists themselves being grouped as “splitters”, “lumpers” or “splimpers” (Dressler 1981). According to Dressler (1981) a “splitter” will assign specific status to everything, based on the theory that they may be different, whereas a “lumper” will declare them the same species if they do not notice a difference. Alternatively, a “splimper” will see important differences within groups that interest them (resulting in splitting of that group), but will lump individuals from groups that are not of direct interest (Dressler 1981).

The bizarre and complicated floral structure of the Orchidaceae has resulted in repeated taxonomic revisions in which groups at various taxonomic levels (i.e., genera and species) have been split or lumped. Taxonomic assessments can often identify extensive or depauperate variation within groups (Cameron et al. 1999; Blaxter 2003) thus, some of the described individuals appear to conform well to evolutionary units (i.e., species), whereas others do not. Whether species are lumped or split can have implications for conservation as some populations of common species may become wrongly conserved, while rarer species worthy of conservation efforts may be ignored because of the choice of taxonomic treatment (Morrison et al. 2009).

Groups of morphologically similar species are common within the Orchidaceae, and several such groups are well-known (e.g., *Cypripedium*, *Dendrobium, Disa*, *Ophrys* and *Phalaenopsis*). *Pterostylis* R.Br. is an Australasian orchid genus in which a high degree of morphological variation confounds the identification and ranking of individual species. As a result, a number of natural groups have been recognized (Dockrill 1992; Jones et al. 1999; Jones and Clements 2002a). One such group is the *Pterostylis longifolia* complex. *Pterostylis longifolia* was first circumscribed by Brown (1810) from New South Wales (Australia). For over 170 years, this taxon was regarded as being widespread and variable.

In 1989, a variable form of Pterostylis occurring throughout New South Wales, Victoria and the Bass Strait Islands was identified as being morphologically distinct from *P. longifolia* and was formally described as *P. tunstallii* (Clements 1989). In 1998, three Tasmanian forms, *P. melagramma, P. stenochila*, and *P. williamsonii*, were segregated from *P. longifolia* (Jones 1998). Consequently, the informal “longifolia” species complex has been recognized for the past 20 years to accommodate the increasing number of morphological variants identified. In 2002, a revision of the subtribe Pterostylidinae placed “longifolia” species into the genus *Bunochilus* D.L. Jones and M.A. Clem. (Jones and Clements 2002a,b) and 4 years later the genus *Bunochilus* was subject to revision. This revision resulted in the recognition of three sections (*Bunochilus, Macrosepalae*, and *Smaragdynae*) and the formal description of 19 new species (*sensu* Jones 2006b). Thus, the total number of “longifolia” species was then 24 (*sensu* Jones 2006b). *Bunochilus* and its sections were not recognized widely (Buchanan 2009) and a recent study by Janes and Duretto (2010) has placed the group within the subgenus *Oligochaetochilus*, section *Squamatae* in an attempt to clarify and simplify the taxonomy.

Irrespective of taxonomic treatment, the “longifolia” complex remains a group of species, that is, confined to south-eastern Australia, including the island of Tasmania. The natural ranges of “longifolia” species overlap frequently. Of the 24 recognized species, 13 have been observed growing sympatrically (Jones 2006b). In spite of frequent sympatric populations, natural hybrids between *P. melagramma* and *P. smaragdyna* are the only confirmed hybrids – morphologically, although several reports of hybrid and potential polyploid individuals have originated from Tasmania (Jones 2006b).

In Tasmania, there are four species within the “longifolia” complex (*sensu* Jones 1998): *Pterostylis melagramma* D.L. Jones (black-stripe greenhood), *P. stenochila* D.L. Jones (green-lip greenhood), *P. tunstallii* D.L. Jones and M.A. Clem. (tunstall's greenhood), and *P. williamsonii* D.L. Jones (brown-lip greenhood). These species have similar morphologies, significant overlaps in ranges and time of anthesis, and are genetically identical in the ITS region (internal transcribed spacer region of nuclear ribosomal DNA) (Janes et al. 2010a). The delimitation of species within the “longifolia” complex (within Tasmania and mainland Australia) not only confounds taxonomists and evolutionary biologists, but has serious implications for conservationists. For example, *P. melagramma* is considered widespread and common throughout Australia; *P. stenochila* and *P. williamsonii* are endemic to Tasmania and locally common in the east; *P. tunstallii* is not endemic, but it is considered endangered within Tasmania because it is restricted to Flinders Island (an island in eastern Bass Strait, between Tasmania and mainland Australia). Furthermore, it is important to determine if hybridization is occurring among these species, creating intermediate types that contribute to indistinguishable morphological boundaries. Thus, confirmation of these taxa as distinct and stable species is required to ensure that proper management techniques are designed.

This study aimed to determine if the “longifolia” species, as defined by Jones (2006b) that are present in Tasmania can be differentiated on the basis of highly polymorphic amplified fragment length polymorphism (AFLP) and chloroplast (cpDNA) polymerase chain reaction (PCR) restriction fragment length polymorphism (PCR-RFLP) markers. AFLP markers have been used widely in orchid studies Hedren et al. (2001), Soliva et al. 2001; Forrest et al. 2004; Mant et al. 2005; Flanagan et al.2006; Tali et al. 2006) and have been shown to be effective in identifying genetic variation within and between populations and closely related species, whereas cpDNA PCR-RFLP markers have been effective in distinguishing interspecific hybrids (Chang et al. 2000) and colonization patterns from seed dispersal (Cozzolino et al. 2003). Herein, we aim to determine population-level genetic structure and species delimitation within the Tasmanian members of the *Pterostylis longifolia* complex.

## Materials and Methods

### Study species

**Figure 1:**
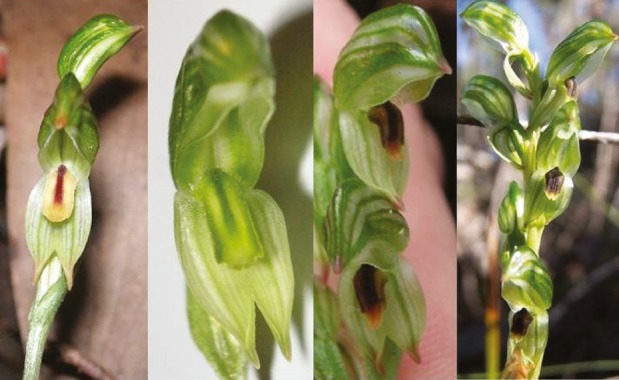
Images of Tasmanian “longifolia” species; from left to right *Pterostylis melagramma, Pterostylis stenochila, Pterostylis tunstallii*, and *Pterostylis williamsonii*.

The *Pterostylis* R.Br. “longifolia” species complex is confined to south-eastern Australia. The complex is demarcated from other Pterostylis species on the basis of several distinguishing features: (1) dimorphic sterile and fertile plants; (2) deflexed lateral sepals with short triangular points; (3) a fully exposed labellum in the set position with three basal lobes and has a distinctive vertical band of color (Jones et al. 1999; Jones 2006a,b). “Longifolia” species, like all Pterostylis*,* are believed to be pollinated through sexual deception (Jones et al. 1999; Jones 2006a) by male members of the superfamily Sciaroidea as Northern (1972) reports pollinators to be gnats. Members of the complex display peak flowering times during the autumn and winter months (Jones et al. 1999; Jones 2006a) and species within the complex are distinguished on the basis of labellum color, length, width, and number of acicular trichomes (Clements 1989; Jones 1998; Jones and Clements 2002a,b) typically. This study focussed on the four Tasmanian representatives of the “longifolia” species (Fig. 1).

### Sample collection

**Figure 2:**
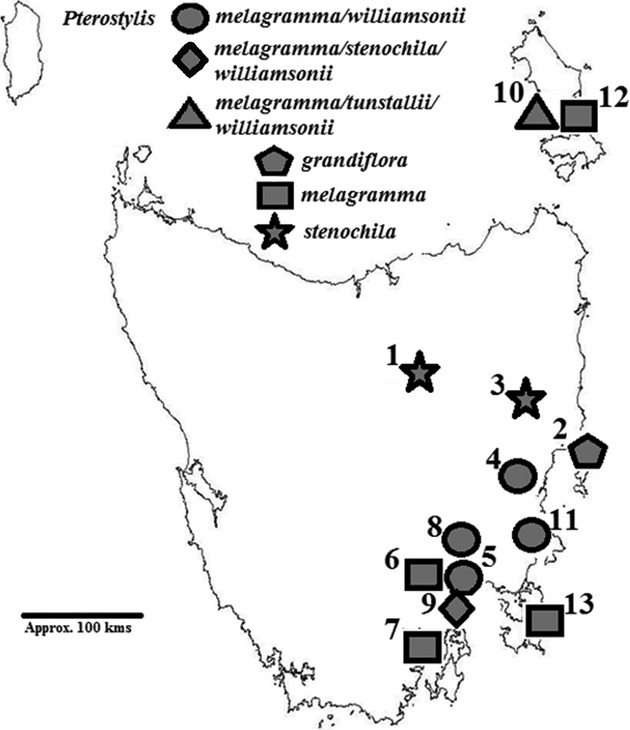
Map of Tasmania showing “longifolia” and *Pterostylis grandiflora* (outgroup) populations that were used in this study. Numbers next to populations indicate site codes (see Table 1). The population circled in the southeast represents multiple collections of *Pterostylis melagramma* from Mt Wellington across the full altitudinal range of the species; see Figure 3 for more detail.

Intensive sampling of “longifolia” species was conducted in eastern Tasmania, including Flinders Island (Fig. 2), during the winter of 2007. *Pterostylis grandiflora* (subgenus Pterostylis, section *Foliosae*) was used as an outgroup to the “longifolia” complex. These samples of *P. grandiflora* were collected from Freycinet Peninsula in eastern Tasmania.

**Figure 3:**
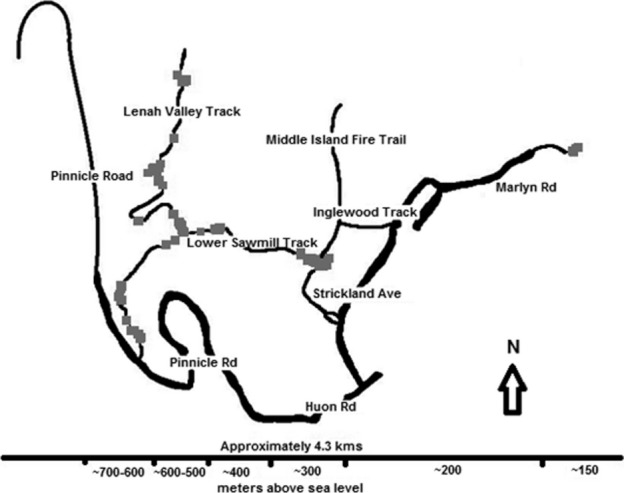
Map of *Pterostylis melagramma* samples (grey squares) collected on Mt Wellington, southeast Tasmania. Thick lines indicate paved roads, thin lines indicate walking tracks and 4WD trails.

One site (Mt. Wellington) was sampled in more detail to enable the detection of fine-scale genetic structure (Fig. 3). The relative positions of each individual and distances between individuals at each site were measured and mapped. Distances between sites were calculated from hand-held GPS readings. Details of sampling sites and sizes are provided in Table 1. A representative voucher specimen of each species at each site was deposited at the Tasmanian Herbarium (HO).

**Table 1:** Details of Tasmanian sites where Pterostylis species were sampled

Species present	Site location	Site No.	Region	Latitude	Longitude	No. individuals used (collected)
*P. grandiflora*	Freycinet	2	NE	−42.1479	148.2867	8 (8)
*P. melagramma*	M-Road	4	NE	−42.3042	147.8754	5 (8)
	Mt. Nelson	5	SE	−42.9277	147.3437	15 (15)
	Mt. Wellington	6	SE	−42.9132	147.2460	98 (177)
	Police Point	7	SE	−43.1850	146.9865	22 (22)
	Risdon	8	SE	−42.8191	147.3195	4 (6)
	South Arm	9	SE	−43.0174	147.5021	4 (4)
	Flinders Island	10	FI (NE)	−40.2045	148.0510	3 (7)
	Thumbs Lookout	11	NE	−42.6096	147.8810	2 (2)
	Vinegar Hill	12	FI (NE)	−40.2023	148.2480	2 (14)
	Waterfall Bay	13	SE	−43.0611	147.9447	5 (6)
*P. stenochila*	Epping Forest	1	NE	−41.7750	147.3217	11 (12)
	Lake Leake	3	NE	−42.0147	147.9556	2 (3)
	South Arm	9	SE	−43.0174	147.5021	10 (10)
*P. tunstallii*	Flinders Island	10	FI (NE)	−40.2045	148.0510	7 (14)
*P. williamsonii*	M-Road	4	NE	−42.3042	147.8754	4 (4)
	Mt. Nelson	5	SE	−42.9277	147.3437	5 (5)
	Risdon	8	SE	−42.8191	147.3195	14 (14)
	South Arm	9	SE	−43.0174	147.5021	7 (8)
	Flinders Island	10	FI (NE)	−40.2045	148.0510	5 (7)
	Thumbs Lookout	11	NE	−42.6096	147.8810	6 (6)
					**Total**	**239 (352)**

FI, Flinders Island; NE, northeast; SE, southeast.

### DNA extraction and PCR-RFLP of cpDNA

Total genomic DNA was extracted from fresh leaf tissue using a DNeasy Plant Mini Kit (Qiagen, Melbourne, Australia). DNA quality was assessed using agarose gel electrophoresis. DNA was quantified using a *Pico*fluor (Turner Designs, California) hand-held fluorometer. A subset of samples representing each species (including the outgroup) from each site were used to detect potential polymorphisms across several cpDNA regions (Table 2). DNA was amplified in a Corbett Research thermocycler according to the protocol of Ebert et al. (2009); with the addition of 2.5 μL of 50% glycerol to each reaction. PCR products were visualized by electrophoresis using a 1% agarose gel in TAE buffer stained with ethidium bromide. Product sizes were estimated by comparison to a 100 bp ladder (Promega, New South Wales, Australia).

**Table 2:** A list of chloroplast primers, sequences, and sources used for cpDNA PCR-RFLP

Marker	Primer sequence	Source
ANU_ChiloCP04	5′- TGATGTTTCTTTCTTTTATCA-3′	Ebert et al. (2009)
	5′- TCATGAATTGACTCTACAAAGGA-3′	Ebert et al. (2009)
ANU_ChiloCP10	5′- TTCTAAAATTTTCAAACCACCT-3′	Ebert et al. (2009)
	5′- GCGTTTCGAACAAATAGAAT-3′	Ebert et al. (2009)
ANU_ChiloCP15	5′- CCATTGGAAATGGAAATAGG-3′	Ebert et al. (2009)
	5′- GGTTTTGGTCCCGTTACTC-3′	Ebert et al. (2009)
ANU_ChiloCP23	5′- AATTTTCACGATTCCTATCCA-3′	Ebert et al. (2009)
	5′- TTTCATTGGAAGAATTGAACC-3′	Ebert et al. (2009)
ANU_ChiloCP37	5′- TTTAGTGTCAGTCTAGAATAACTGG-3′	Ebert et al. (2009)
	5′- GCATCAAAGAGCTAAATGAGA-3′	Ebert et al. (2009)
ANU_ChiloCP38	5′- GGGGATCAGTTGGATCTTTG-3′	Ebert et al. (2009)
	5′- CCAATTTGACCCCCTACAAG-3′	Ebert et al. (2009)
ANU_ChiloCP41	5′- TGCCAAACAGGTGAAGTACA-3′	Ebert et al. (2009)
	5′- AACACGATACCAAGGCAAAC-3′	Ebert et al. (2009)
ANU_ChiloCP45	5′- TGGCATTAGCATCACAAAGA-3′	Ebert et al. (2009)
	5′- GGTTTCTGCGGATATGGAAT-3′	Ebert et al. (2009)
ANU_ChiloCP68	5′- TCAGCGGGGGAATAGAAAT-3′	Ebert et al. (2009)
	5′- GATAGGAACAATGGCGAAGC-3′	Ebert et al. (2009)
*trn*D	5′-ACCAATTGAACTACAATCCC-3′	Demesure et al. (1995)
*trn*T	5′-CTACCACTGACTTAAAAGGG-3′	Demesure et al. (1995)
*trn*L	5′-GGTTCAACTCCCTCTATCCC-3′	Taberlet et al. (1991)
*trn*F	5′-ATTTGAACTGGTGACACGAG-3′	Taberlet et al. (1991)
*trn*M	5′-TACCTACTATTGGATTTGAAC-3′	Cheng et al. (2005)
*trn*V	5′-GCTATACGGGCTCGAACC-3′	Cheng et al. (2005)
*trn*S	5′-GAGAGAGAGGGATTCGAA-3′	Demesure et al. (1995)
*trn*FM	5′-CATAACCTTGAGGTCACGGG-3′	Demesure et al. (1995)

Successfully amplified cpDNA products were digested with each of the following restriction enzymes: *Alu* I, *Xmn* I, *Hinf* I, *Taq*^*a*^ I, *Ssp* I, and *Mse* I (New England Biolabs, Massachusetts). Restriction digests for *Alu* I, *Xmn* I, *Hinf* I, *Ssp* I, and *Mse* I contained 2 μL of NEB Buffer 2 (New England Biolabs, Massachusetts), 0.5 μL of restriction enzyme, 5 μL PCR product and 5 μL H_2_O, and were incubated overnight at 37°C. Restriction digests for *Taq*^*a*^ I contained 2 μL of NEB Buffer 3 (New England Biolabs, Massachusetts), 2 μL 100 μg/mL BSA (Bovine Serum Albumin), 0.25 μL of enzyme, 2.5 μL PCR product and 2.5 μL H_2_O, and were incubated at 65°C for 4.5 h. Restriction digests were assessed by electrophoresis using 2% agarose gels in TAE buffer stained with ethidium bromide. Product sizes were estimated by comparison with a 100 bp ladder (Promega, New South Wales, Australia).

### AFLP fragment analysis

A modified version of the AFLP method (originally described by Vos et al. 1995) with simultaneous restriction and ligation of DNA was used (McKinnon et al. 2008). Pre–amplification using *Mse* I and *Eco* retention index (RI) adapters and selective amplification PCRs were conducted in a Corbett Research thermocycler (Corbett Research, Sydney, Australia). All PCR conditions followed those of Vos et al. (1995). For the selective amplification, 12 different primer combinations were tested on 12 “longifolia” samples. Three combinations were chosen after screening: AAG/CGG, AGA/CAG, and AGA/CGG. Fragment separation and detection were performed using a CEQ 8000 Genetic Analysis System 8.0.52 (Beckman Coulter, Gladesville, Australia). Fragments (100–590 bp) were scored as presence/absence (binary) scores (maximum bin width of 1.00, Y-threshold of 0.00). Due to the high number of fragment peaks detected, each sample was checked manually and the following were excluded from further analysis: (1) samples that failed to generate readable profiles; (2) samples with poorly defined fragment peaks; (3) overlapping bins (the software occasionally generated bins of the same size, but with overlapping size ranges) and; (4) bins containing fragments from fewer than 10 samples (thus a 4.3% threshold). To calculate repeatability between runs, one sample was represented twice (independently extracted, restricted, ligated and amplified) in each run. The fragment data from the duplicated samples were compared and the error rate was expressed as a percentage of the total number of bands. In addition, two negative controls (i.e., no DNA) were included in each digest, ligation, pre-amplification, selective PCR, and sequencing run to detect possible contamination.

### Genetic diversity analyses

In all analyses, different “longifolia” species at the same site were treated as separate “populations” (this study involved 21 populations including outgroup). In addition, the different “longifolia” species at a site were grouped together (irrespective of species) and called “sites” (13 sites including outgroup) for nested analyses of molecular variance (AMOVA) analyses. AFLP profiles were analyzed using HICKORY (Holsinger et al. 2002) and AFLP-SURV (Vekemans 2002) to determine the level of genetic differentiation between populations (*F*_*ST*_) and to obtain an estimate of the inbreeding coefficient (*F*_*IS*_ or ƒ).

Dominant markers, such as AFLP, prevent the direct estimation of inbreeding coefficients (*F*_*IS*_ or ƒ) because heterozygosity remains undetected (Holsinger et al. 2002; Vekemans 2002). As a result, population structure analyses are weakened because there is no estimate of *F*_*IS*_ or ƒ. HICKORY estimates an *F*_*ST*_ analog (designated *Θ*^*II*^) from dominant markers, whereas accounting for the uncertainty associated with the inbreeding coefficient (ƒ). Four models are fitted to the data: (1) both *Θ*^*II*^ and ƒ are unknown and ≥ 0 (full model); (2) *Θ*^*II*^ is unknown but no inbreeding occurs (ƒ = 0 model); (3) there is no genetic structure but ƒ is unknown (*Θ*^*II*^ = 0 model); (4) ƒ is selected from a prior distribution without generating a posterior distribution of ƒ (ƒ–free model). All HICKORY analyses were performed using default values for sampling and chain length parameters (burn-in = 5000, sample = 100,000, thin = 20).

Pairwise *F*_*ST*_ values among populations were computed with the non-uniform prior distribution Bayesian estimator in AFLP-SURV. Analyses were performed assuming Hardy–Weinberg equilibrium initially, and then deviation from Hardy–Weinberg equilibrium was accounted for in accordance with ƒ values estimated by HICKORY. Each time, the data were subjected to 999 permutations. Pairwise *F*_*ST*_ values were used in Principal Coordinates Analyses (PCoA) using GenAlEx 6 (Peakall and Smouse 2006). These analyses allowed for the relationships between populations to be visualized.

Genetic distance values between pairs of “longifolia” individuals were calculated in PAUP*4.0b (Swofford 2001) using Nei and Li (1979) genetic distance. The individual-based “longifolia” pairwise distance matrices were imported into GenAlEx 6 for hierarchical AMOVA. When performing AMOVA in GenAlEx 6 a pairwise individual by individual genetic distance matrix is created using a binary distance option for AFLPs such that genetic distances amount to a tally of the band differences between samples (Flanagan et al. 2006). The total genetic variation is partitioned at three levels – within populations (Phi-PT), among populations within taxa (Phi-PR), and among taxa (Phi-RT) (Flanagan et al. 2006). Tests for significant departure from the null hypothesis of “no genetic structure” at each hierarchical level (i.e., individuals – taxa – sites; individuals – populations – taxa; and regionally for both southeast and northeast Tasmania, individuals – populations – taxa) were performed using 999 random permutations of the raw data.

### Analysis of population genetic structure

The number of groups of genetically similar individuals (*K*) in the “longifolia” complex, and the affinities of individuals to each group, were determined using the Bayesian clustering algorithm employed by TESS version 1.2 (Chen et al. 2007). TESS version 1.2 assumes no prior population groupings using the admixture model (default interaction parameter of 0.6, burn-in of 5000 repetitions, 10,000 MCMC repetitions) thus, species delimitation could, theoretically, be inferred from the assignment of individuals to a cluster, irrespective of geographical location or phenotype. One hundred independent runs for each value of *K* were performed, and results were interpreted using the 20 runs with the highest likelihood. The estimated *K* was determined by comparing the log probability of data at different values of *K* (from *K* = 1 to *K* = 9 – at which point stationarity had been reached) and the Δ*K* method described by Evanno et al. (2005).

The values of *K* at which the mean likelihood and Δ*K* were maximized were interpreted as the “best” *K*. The *LargeK Greedy* algorithm (using a random input order, the G' pairwise matrix similarity statistic and 10,000 permutations) of CLUMPP (Jakobsson and Rosenberg 2007) was used to obtain a single, optimal alignment. Results were visualized using DISTRUCT version 1.1 (Rosenberg 2004).

## Results

### Levels of genetic diversity in “longifolia” species

A total of 28 samples were used to screen the 78 cpDNA primer and restriction enzyme combinations for potential polymorphism. Thirteen combinations of primers and enzymes exhibited fragment variation, 10 of which distinguished the outgroup, *P. grandiflora,* but were not polymorphic within the “longifolia” complex. Three combinations identified variation within the “longifolia” complex, but all these mutations were found only in a small number of samples. Overall, few polymorphisms were detected and they were not useful for discriminating species within the “longifolia” complex nor for distinguishing sites thus, further cpDNA work was abandoned. A summary of the cpDNA variation is provided in Table 3.

**Table 3:** Summary of results from the cpDNA RFLP screening on Tasmanian “longifolia” species

Primers	Restriction enzyme	Variation identified
*trnD – trnT*	*Alu*I	Outgroup
*trnL – trnF*	*Taqa*I	Outgroup
*trnL – trnF*	*Xmn*I	Outgroup
*trnL – trnF*	*Hinf*I	Outgroup
*trnM – trnV*	*Hinf*I	Outgroup
*trnS – trnFM*	*Hinf*I	Outgroup
8	*Alu*I	Four *Pterostylis melagramma*, one *Pterostylis williamsonii* from Site 5
8	*Hinf*I	Outgroup
12	*Hinf*I	One *Pterostylis stenochila* from Site 3, one *P. williamsonii* from Site 8
38	*Mse*I	Two *P. stenochila* from Site 1, one *P. stenochila* from Site 9
38	*Taqa*I	Outgroup
40	*Alu*I	Outgroup
40	*Xmn*I	Outgroup

For each of the 231 samples, 247 unambiguous AFLP bands were scored, of which 228 were polymorphic (92%). No markers were identified as species–specific within the “longifolia” complex. The levels of variation observed between sample replicates were estimated by dissimilarity matrix and ranged from 0 to 2% and averaged 1.14%, indicating a high degree of repeatability between runs. No pairs of “longifolia” samples were found that were more similar to one another than the average error rate of 1.14%, indicating that, in all likelihood, no clonal ramets had been sampled. Thirteen samples were identified as having very close relationships (within 5%) within the Epping Forest *P. stenochila* population and the Mt. Wellington *P. melagramma* population. Five samples from the Pterostylis outgroup, *P. grandiflora*, were within the 2% range, indicating the possibility of clonal samples.

The Tasmanian “longifolia” species complex was weakly structured according to HICKORY estimates of the levels of differentiation, as indicated by a low *Θ*^*II*^ (< 0.05 for all Bayesian models). The full model, which simultaneously estimates *Θ*^*II*^ and the inbreeding coefficient ƒ, best fitted the data by having the lowest Deviance Information Criterion value (DIC = 14070), suggesting that inbreeding was high in “longifolia” populations (ƒ = 0.8, SD = 0.18). However, there was little difference in the DIC values between the full model and the next best model ƒ = 0 (14070 vs. 14078, respectively). The level of genetic differentiation within the “longifolia” complex estimated by AFLP-SURV using the *F*_*ST*_ statistic was substantially higher than that obtained from HICKORY (*F*_*ST*_ = 0.18 vs*. Θ*^*II*^ = 0.004). Neither HICKORY's *Θ*^*II*^ nor AFLP-SURV's *F*_*ST*_ values appeared to accurately reflect the patterns obtained from population structure and PCoA analyses (see below). Thus, the contrasting results obtained in the different analyses, coupled with conflicting population structure results suggest that these estimates of genetic differentiation have little support.

AMOVA tests using “populations” (excluding the outgroup) indicated that 69% of the total genetic variation was distributed among individuals, 24% was found among populations of a species and only 7% of the total genetic variation was distributed among taxa (Phi–PR 0.308, Phi-PT 0.254 and Phi-RT 0.072, respectively; *P* = 0.01). A second nested AMOVA, using “sites”, indicated that 71% of the total genetic variation was distributed among individuals, 5% was distributed among species within a site and 24% of the total genetic variation was distributed among sites (Phi-PR 0.290, Phi-PT 0.065 and Phi-RT 0.242, respectively; *P* = <0.005). Furthermore, AMOVAs performed at a regional level (i.e., northeast Tasmania and southeast Tasmania) indicated that the genetic diversity of “longifolia” samples from southeast Tasmania was partitioned in the following way: 83% of the genetic variation was distributed among individuals, 11% among populations within a species and 6% among taxa (Phi-PR 0.173, Phi-PT 0.122 and Phi-RT 0.058; *P* = 0.01). In contrast, “longifolia” samples from northeast Tasmania showed 62% of the genetic variation distributed among individuals, 29% among populations and 9% among taxa (Phi-PR 0.383, Phi-PT 0.319 and Phi-RT 0.094, respectively; *P* = 0.01).

**Figure 4:**
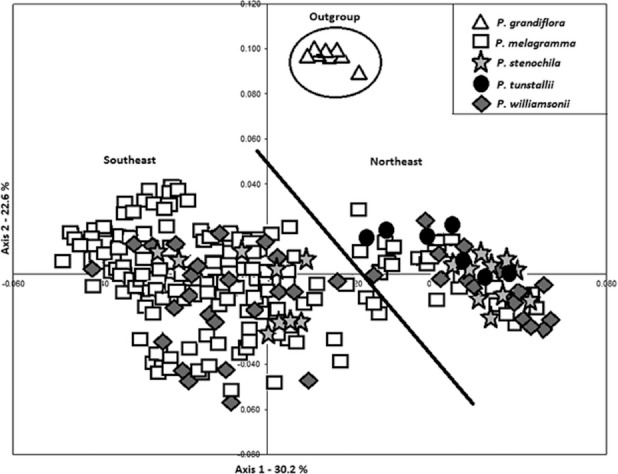
Principle coordinates analysis of “longifolia” complex individuals. The diagonal line highlights the partition between individuals from southeast Tasmania (left) and those from northeast Tasmania, including Flinders Island (right). Axes 1 (horizontal) and 2 explain 30.2% and 22.6% of the total variation, respectively.

**Figure 5:**
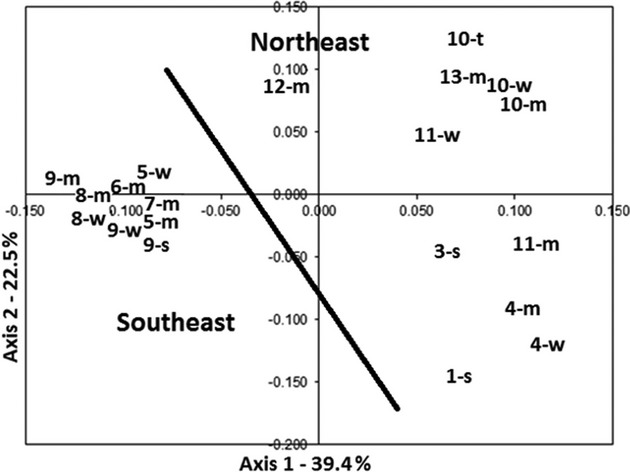
Principle coordinates analysis of “longifolia” populations. Populations are grouped by site (for each species). The diagonal line highlights the partition between populations from southeast Tasmania (left) and those from northeast Tasmania, including Flinders Island (right). Axes 1 (horizontal) and 2 explain 39.4% and 22.5% of the total variation, respectively. Numbers indicate sites (refer to Table 1) and lower case letters refer to species (m – *P. melagramma*, s – *P. stenochila*, t – *P. tunstallii*, w – *P. williamsonii*)

A PCoA showed that while the outgroup (*P. grandiflora*) formed a discrete cluster the “longifolia” individuals were poorly separated from one another with no clear demarcation of species (Fig. 4). A subtle partition was found between “longifolia” individuals from the northeast of Tasmania and those from the southeast of Tasmania (Fig. 4). The samples from Waterfall Bay (Tasman Peninsula in southeast Tasmania) grouped with the cluster of individuals from the northeast. A PCoA of “longifolia” populations (Fig. 5) revealed a similar pattern, although populations from the northeast of Tasmania appeared to have higher levels of genetic differentiation compared with populations from the southeast of Tasmania.

### Population genetic structure within the “longifolia” complex

**Figure 6:**
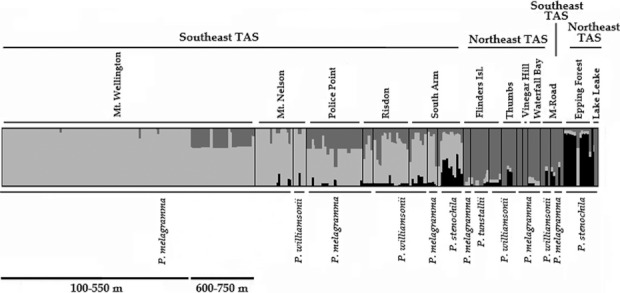
Histogram of averaged assignment probabilities (calculated by TESS, averaged by CLUMPP and visualized using DISTRUCT). Each vertical bar represents an individual and its assignment proportion into one of three clusters. Individuals are arranged by species (below) and sites (above). Sites are further divided into regions (above) and altitudes are provided for the *P. melagramma* samples from Mt. Wellington (below).

The optimal number of clusters that explained the genetic variation in the data was identified as *K* = 3 when using the methods of Evanno et al. (2005) and others (Francois et al. 2006; Chen et al. 2007; Falush et al. 2007) (Fig. 6). When *K* = 3, there was clear distinction between “longifolia” individuals sampled from the northeast and southeast of Tasmania, with one exception: the Waterfall Bay population of *P. melagramma* collected from southeast Tasmania on the Tasman Peninsula, which consistently grouped with those from the northeast. At higher levels of genetic structure (*K* > 3), this trend remained consistent, with samples from northeast Tasmania and Waterfall Bay being genetically differentiated from those of southeast Tasmania, as was *Pterostylis stenochila*. Within southeast Tasmania, there appeared to be evidence for a genetic cline in samples of *P. melagramma* from Mt. Wellington over its altitudinal range. For example, samples collected at the upper altitudinal range (600–800 m) had a higher proportion of the “dark grey” genotype than samples from lower altitudinal sites (Fig. 6).

## Discussion

The results presented herein show a lack of genetic distinction between “longifolia” species, but reveal strong genetic structure. Herein, the use of a powerful discriminating tool, such as AFLP, suggests that the Tasmanian “species” (*sensu* Jones 2006b) of the “longifolia” complex are not separate species or even taxa. However, the genetic structure evident shows clear patterns of regional distinction. Typically, species fit a particular species concept, for example, the ecological species concept (i.e., species have well-defined, discrete fundamental niches), the evolutionary species concept (i.e., species share a common ancestor but have sufficient divergence to be considered separate species), and/or the biological species concept (i.e., a group of naturally interbreeding populations that are reproductively isolated from other such groups) (Coyne and Orr 2004). However, the Tasmanian “longifolia” species did not fit any of these concepts. The ecological species concept can be dismissed because the species could not be separated along ecological gradients into distinct niche spaces (Janes et al. 2010b). In the present study, AFLP profiles did not identify sufficient genetic variation to partition the species, effectively nullifying the evolutionary species concept. On the basis of the results presented herein, and reports of “intermediate” types (Jones 2006b), it appears that the Tasmanian “longifolia” species do not fit the biological species concept either. Explanations for this apparent poor fit include: (1) the AFLP technique is ineffective in delimiting orchid species; (2) taxonomists have erroneously treated morphotypes or ecotypes as species; (3) the species are subject to long distance dispersal events and subsequent inter-specific hybridization; and/or (4) populations of the species are in the process of expanding their ranges from historic glacial refugia and have incomplete reproductive barriers. Reasoning for and against these hypotheses will be discussed in detail.

*Explanation 1 – The AFLP technique is ineffective in delimiting orchid species*. This technique has been used extensively in studies dealing with taxonomically difficult plant species (Krauss and Peakall 1998; McKinnon et al. 1999; O'Hanlon et al. 1999; Pfeifer et al. 2006), and has successfully elucidated taxonomic complexity and hybrids in several orchid genera (Soliva et al. 2001; Cozzolino et al. 2006; Flanagan et al. 2006). The AFLP profiles from *Corybas* species that were generated in our laboratory, using the same primer pairs that were used in the present study, successfully delimited species (data not presented). Furthermore, the AFLP revealed genetic differentiation between populations. Thus, the AFLP technique should have sufficient power to identify genetically isolated/morphologically distinct species within Pterostylis.

*Explanation 2 – Taxonomists have erroneously treated morphotypes or ecotypes as species*. Taxonomic over-splitting is considered to occur frequently within the Orchidaceae (Dressler and Dodson 1960; Hopper and Brown 2004; Pillon and Chase 2007). Primarily, over-splitting in orchids is thought to stem from the sheer diversity present within the group as morphological species classifications are often based on characters that are later found to exist in unrelated groups (Dressler 1981), resulting in further taxonomic investigation to highlight characters that truly distinguish the groups. Such investigations may result in several new taxonomic combinations, which further complicate the overall circumscription of what may already be considered a taxonomically complex group. Furthermore, increased numbers of molecular phylogenetic studies are proving that many taxa are invalid because they lack sufficient genetic variation (thus nullifying phylogenetic species concepts also) (Pillon and Chase 2007). However, over-splitting may also be the result of the popularity attributed to orchids and people's enthusiasm to draw attention to interesting morphotypes.

In the case of the “longifolia” complex, the division of *P. longifolia* into four taxa has arisen from two authorities (Clements 1989; Jones 1998) and the characters used are all based on subtle shifts in morphology (i.e., the color and size of the labella). *Pterostylis melagramma, P. tunstallii* and *P. williamsonii* all possess labella of varying shades of brown to golden-brown while *P. stenochila* has a green labellum. The subtle morphological differentiation between *P. melagramma, P. tunstallii*, and *P. williamsonii* may actually represent intermediate forms (i.e., morphotypes or ecotypes) which is indicative, typically, of hybridization (Coyne and Orr 2004).

*Explanation 3 – The species are subject to long distance dispersal events and subsequent inter-specific hybridization*. This hypothesis gains some support from reports of putative hybrids between *P. melagramma* and *P. stenochila* (Jones 2006b) and from the population genetic structure analysis in which three distinct groups were revealed. Although inter-specific hybridization is common in orchids (Dressler 1981; Arduino et al. 1996; Cozzolino et al. 2006), it seems more common for sexually deceptive species, such as Pterostylis, to have a specific pollinator species (i.e., one insect pollinator species to one orchid species; see reports from *Caladenia* Phillips et al. 2009; *Chiloglottis* Mant et al. 2005; *Ophrys* Ayasse et al. 2000), a relationship, which is believed to result in the reproductive isolation of many closely related orchid species (Dressler 1981; Schiestl et al. 1999). However, hybridization is unlikely to be the result of long distance pollen dispersal in this instance because Pterostylis are presumably pollinated by gnats. Research into the distances traveled by the more common pollinators (bees and thynnine wasps) of sexually deceptive orchids indicates that travel distances are relatively short (cm to m) (Peakall and Schiestl 2004; Wong et al. 2004). Given that gnat species are significantly smaller than bees and thynnine wasps one would expect them to travel even shorter distances typically. Thus, it seems unlikely that extensive hybridization, as a result of long distance pollen-mediated gene flow between species, is responsible for the observed pattern in genetic structure seen here.

It is possible that hybridization is the result of long distance seed dispersal and subsequent short distance pollen dispersal. Orchid seed is minute and, although seed dispersal is often highly localized within populations, some seed can enter the air column and disperse over long distances (Brundrett et al. 2003; Trapnell et al. 2004). The identification of populations of *P. stenochila* that had a high proportion of genetic assignment from populations of other species suggested that seed may have been dispersed over considerable distances. However, one would also expect the genetic structure profiles of *P. tunstallii* and *P. williamsonii* to have somewhat equal proportions of parental profiles if they were the product of hybridization between *P. melagramma* and *P. stenochila*. The genetic structure of *P. tunstallii* and *P. williamsonii,* however, did not follow a typical hybridization pattern and *P. stenochila* remained the most distinct taxon in terms of genetic differentiation and structure, whereas *P. melagramma, P. tunstallii*, and *P. williamsonii* were indistinguishable on the basis of AFLP. As such, it seems more probable that, in this instance, the subtle morphological variation between “types” has been exaggerated, whereas the genetic structuring of “longifolia” populations conforming to geography suggests that there may have been some sort of barrier to gene flow.

*Explanation 4 – Populations of the species are in the process of expanding their ranges from historic glacial refugia and have incomplete reproductive barriers*. The distribution of genetic variation in the “longifolia” complex may indicate the existence of climatic refugia for “longifolia” species. Genetic variation at a regional scale that does not necessarily correlate with circumscribed taxa, have been explained by the presence of historical climatic refugia for other orchid species, such as *Anacamptis palustrus* (Mediterranean) (Cozzolino et al. 2003) and the *Cypripedium parviflorum* complex (North America) (Wallace and Case 2000).

The high frequency of endemic species in the southeast of Tasmania (Kirkpatrick and Brown 1984) has long been used as an argument to support the existence of a large glacial refugium in that area (Kirkpatrick and Fowler 1998). Evidence from chloroplast DNA from *Eucalyptus* (McKinnon et al. 2001, 2004) and *Nothofagus* (Worth et al. 2009) further supports the idea. Kirkpatrick and Fowler (1998) proposed that small glacial refugia existed within the southeast of Tasmania and that larger refugia existed at the northern extremes of the island and former Bassian Plain (now mainly submerged and represented by the Bass Strait Islands). During the early Holocene, eucalypt species are thought to have migrated mainly out of northern and southern refugia to colonize most of the island (McKinnon et al. 2004). Possibly, as a result of long-term isolation in distinct refugia, eucalypt species within Tasmania show a high degree of endemism coupled with distinct regional genotypes (e.g., northern and southern) (McKinnon et al. 2004). A similar scenario may apply to Pterostylis species.

The hypothesis of Pterostylis species with incomplete reproductive barriers expanding their ranges is supported by: (1) the restricted distribution and endemism of *P. stenochila* and *P. williamsonii* in relation to the more widely occurring *P. melagramma*; (2) the distinction between the northeastern and southeastern populations within the complex; and (3) the restricted distribution of *P. tunstallii* on Flinders Island and in Victoria. From these data, it may be conjectured that *P. melagramma* persisted through the last glacial maximum in several refugia, occurring in southern Victoria, the Bass Strait islands, northern Tasmania, and southeastern Tasmania. During the glacial maximum, limited gene flow may have led to genetic drift and inbreeding increasing divergence between populations in different refugia. Following this period of isolation and potential inbreeding, a relatively rapid period of re-colonization via occasional long distance dispersal, potentially with the assistance of migrating birds (Arditti and Ghani 2000), and subsequent genetic introgression may have resulted in great morphological and ecological variation in *P. melagramma*.

This AFLP-based study has shown the Tasmanian “longifolia” complex to be a group of genetically very similar populations. The lack of species-specific markers and genetic differentiation of morphological species using a powerful discriminating technique like AFLP raises questions relating to the specific status of the “longifolia” taxa. The lack of differentiation at the species level suggests that morphological variants have been incorrectly described as species. Genetic structuring at a regional scale suggests that geographic isolation has occurred at some point and resulted in a level of genetic divergence. These regional populations may be in the process of converging following isolation during the last glacial event.

The evidence gained from this research suggests that the “longifolia” complex in Tasmania may best be treated as a single taxon *sensu* W.M. Curtis (1979) with several “varieties” of similar genetic makeup. This scenario has important consequences for the conservation status within Tasmania and, potentially, the entire complex in southeastern Australia if similar results were found across the entire “longifolia” complex range.

Although the re-instatement of *P. longifolia sensu lato* W.M. Curtis across the “longifolia” complex range is unlikely based on this data alone, such a decision would effectively sink approximately 23 species, thereby reducing the complex to a single, widespread and variable species that is not under immediate threat of extinction. A taxonomic review of this magnitude would certainly lessen taxonomic confusion. However, several of the morphotypes are quite clearly rarer than others and may be contributing important genetic variation into the populations and facilitating speciation, thereby raising issues relating to the conservation of organisms below the rank of species. At present, approximately seven “longifolia” species are under State and/or National protection (Environment Protection and Biodiversity Conservation Act 1999). Thus, in order to continue to facilitate gene flow between “varieties” (i.e., allowing for any potential speciation events in the future), it may be necessary to attempt to formally conserve specific populations. Australian threatened species guidelines currently allow for the formal conservation of native flora below the level of subspecies, but only if the taxon is narrowly defined in terms of taxonomy and geography and only if there is a special need. Thus, the conservation of morphotypes is likely to remain a difficult and unpopular process until further research is conducted in this area to facilitate a greater understanding of the evolutionary processes at work within species complexes, such as the greater “longifolia” complex.
